# A new attempt to treat coronal plane fractures of the elbow joint with salvage via an anterior approach

**DOI:** 10.1186/s12893-022-01706-9

**Published:** 2022-07-04

**Authors:** Qiyu Jia, Xiangxiang Li, Jing Zhan, Dongsheng Chen, Kai Liu, Yingbo Wang, Aihemaitijiang Yusufu, Chuang Ma

**Affiliations:** 1 Department of Microrepair and Reconstruction, The First Affiliated Hospital of Xinjiang Medical University, Urumqi, Xinjiang China; 2 Department of Trauma Surgery, Yanzhou District People’s Hospital, Jining, Shandong China; 3 Department of Gynecology, Yanzhou District People’s Hospital, Jining, Shandong China; 4 College of Chemical Engineering, Xinjiang Normal University, Urumqi, Xinjiang China

**Keywords:** Anterior approach, Coronal plane, Elbow fracture, Internal fixation, Treatment

## Abstract

**Background:**

Existing approaches for treating elbow fractures include lateral, medial, anterior and posterior approaches, though the anterior approach is often not chosen by surgeons to avoid damage to important nerves and blood vessels. However, the anterior approach has unique advantages. The purpose of this study was to report outcomes of 38 patients with coronal plane elbow fractures treated through the anterior approach.

**Methods:**

We retrospectively analyzed 38 cases of coronal plane elbow fracture treated through an anterior approach at our institution between March 2015 and July 2019. The length of the surgical incision, operation time, and postoperative complications were recorded. The range of flexion, extension, and rotation of the affected elbow and the healthy elbow were collected at follow-up. Functional outcomes were evaluated using the Mayo Elbow Function Score (MEPS).

**Results:**

All 38 patients were followed up for a mean of 21.26 months (range 12–36 months). Intraoperatively, the mean surgical incision length was 8 ± 2 cm and the mean operative time was 123 ± 59 min. At the final follow-up, solid osseous union was confirmed for all coronal plane elbow fractures. The mean elbow flexion arc was 129 ± 7°, and the extension arc was 9 ± 6°. The mean pronation arc was 83 ± 3°, and the supination arc was 80 ± 3°. The mean MEPS was 90 ± 8 points, with 18 excellent cases and 20 cases of excellent and good results, respectively. In 31 cases, there was no significant difference in elbow extension, flexion, or pronation between the single-fracture and healthy elbows (*P* > 0.05), though the arc of supination was slightly worse than that of the healthy elbow (*P* < 0.05). VAS pain scores before the operation, at three months after the operation, and during follow-up were compared, and pain was significantly reduced after treatment (*P* < 0.05). Two patients experienced transient postoperative median nerve paralysis, from which they recovered within three months. One patient had mild heterotopic ossification and was not treated because it did not affect the function of the elbow joint. All patients returned to work and were satisfied with the treatment.

**Conclusion:**

The anterior approach has the benefits of simplicity, safety, minimal invasiveness, excellent exposure, and satisfactory prognosis for coronal plane elbow fracture.

## Background

Due to the specific nature of intra-articular fractures, treatment of elbow fractures must conform to the criteria of anatomical reduction, and good surgical exposure and firm fixation are necessary. An optimal procedure should be performed in a way that minimizes soft tissue stripping without compromising fracture visualization and reduces the risk of elbow stiffness [1, 2], which is closely related to the choice of surgical approach. At present, available approaches for the treatment of elbow fractures include anterior, lateral, medial, and posterior approaches [3–5]. Of these, lateral, medial, and posterior approaches are more commonly used, and patients with the complex “terrible triad” injury are often treated with a combined approach. In contrast, the anterior approach for the elbow is less frequently chosen by surgeons due to its anatomical peculiarities, with nerves and vessels intertwined in the operative field.

Moreover, the anterior approach has unique advantages. It allows for reduction and fixation of fragments and repair of the anterior joint capsule through direct sight, providing more options for internal fixation, and is gradually being promoted in the treatment of coronoid process fractures [6, 7]. Indeed, in the last years, the anterior approach to the elbow has been considered as a valid alternative for fractures of the distal humerus, coronoid and radial head [8–10].

Radial head fractures and partial distal humeral fractures that occur in the coronal plane of the elbow, as well as the terrible triad, can be well treated with an anterior approach; there are even demonstrate unique advantages, which have rarely been reported in the literature [11]. Although outcomes of treatment of a single elbow fracture through an anterior approach has been described, a series of fractures in the coronal plane has not been summarized and reported.

The aim of this study was to present treatment of a series of fractures in the coronal plane of the elbow joint by using an anterior approach and to analyze its effectiveness and unique advantages.

## Methods

After receiving approval from our Institutional Review Board, we retrospectively analyzed 38 consecutive patients (25 males, 13 females; mean age, 32.8 years; range, 14–69 years) who had undergone open reduction and internal fixation through an anterior approach in our hospital for the treatment of coronal plane elbow fractures between March 2015 and July 2019 (Table 1).

**Table 1:** Baseline information

Case	Sex	Age (years)	Side	Fracture classification	Elbow dislocation	Operation time (m)	Incision length (cm)	Union time (w)
1	M	14	L	13B3.2	No	70	5	14
2	M	29	L	R-M: II; mason: IV	Yes	190	6	18
3	M	15	R	13B3.2	No	135	6	13
4	M	24	R	R-M: II	No	150	8	14
5	M	23	L	R-M: II	No	180	8	12
6	M	27	R	R-M: II	No	150	6	16
7	M	40	L	R-M: II	No	105	7	14
8	M	33	R	R-M: II	No	150	8	13
9	M	31	L	R-M: II	No	133	7	15
10	M	34	L	R-M: II	No	136	6	13
11	M	37	L	R-MII; mason: IV	Yes	310	12	14
12	F	19	L	R-M: III	No	120	10	13
13	M	32	R	mason: II	No	180	6	15
14	M	23	R	mason: II	No	105	6	14
15	F	37	L	mason: III	No	75	10	15
16	F	56	L	R-M: II	Yes	70	6	14
17	F	52	L	R-M: II	No	165	10	14
18	M	29	R	R-MII; mason: IV	Yes	180	6	16
19	F	48	R	mason: II	No	80	6	16
20	F	54	R	R-MIII; mason: IV	Yes	180	10	15
21	M	57	L	R-MIII; mason: III	Yes	150	8	15
22	M	34	L	R-MII; mason: IV	Yes	300	6	16
23	M	29	L	R-MIII; mason: III	Yes	150	10	16
24	M	28	R	R-M: II	No	77	7	12
25	M	32	R	mason: III	No	90	6	16
26	F	21	R	mason: III	No	60	10	12
27	M	29	R	R-M: III	No	65	12	12
28	F	39	L	mason: III	No	95	6	15
29	F	69	L	13B3.2	No	85	8	14
30	F	31	L	13B3.1	No	100	10	15
31	M	28	L	13B3.1	No	75	8	13
32	M	16	R	13B3.2	No	65	7	15
33	M	16	L	13B3.2	No	65	6	15
34	F	21	R	13B3.2	No	80	10	16
35	F	22	L	13B3.3	No	120	10	14
36	M	44	L	R-M: II	No	75	7	15
37	F	26	R	R-M: III	No	80	8	14
38	M	47	R	R-M: II	No	80	8	16

*y* year, *w* week, *M* male, *F* female, *L* left, *R* right

The right extremity was injured in 17 patients and the left in 21. The primary goal of surgical fixation was to obtain a stable joint that permitted early movement. Each fracture was classified with AO/OTA classification for distal humerus fracture, Regan-Morrey classification for coronoid process fractures and Mason classification for radial head fractures. Regan-Morrey type I and Mason type I fractures with good elbow stability or fractures without significant displacement were excluded. The inclusion criteria were as follows: a Regan-Morrey type II or type III coronoid process fracture; Mason type II, III, and IV radial head fractures; and distal humeral fractures included in AO types 13B 3.1, 13B 3.2, and 13B 3.3. According to these classifications, there were 16 cases of Regan-Morrey type II, 6 of Regan-Morrey type III, 3 of Mason type II, 6 of Mason type III, and 5 of Mason type IV; for AO type, there were 2 cases of type 13B3.1, 6 of type 13B3.2, and 1 of type 13B3.3. All 38 patients qualified for the final evaluation.

The mechanism of injury included 26 cases of falling on flat ground while walking, 8 cases of falling from a height, 3 cases of a traffic accident, and 1 case of heavy object impact. There were 7 cases of coronoid process with radial head fracture. The patients received initial trauma control in the Emergency Department.

All patients had closed injuries with no neurovascular complications. All 38 elbows were treated surgically at a mean of 4.3 days (range 2–12 days) after the initial injury. The specific indications for surgical intervention include displaced intra-articular fracture and elbow instability within a functional arc of motion after closed reduction.

Preoperative radiological examinations were routinely performed to determine the exact location of fracture in the coronal plane. All patients underwent preoperative CT and 3D reconstruction of the elbow joint to clarify the fracture type and the displacement direction of the fracture fragment to clarify the diagnosis and design the surgical plan.

### Surgical technique

All patients were treated with nerve block anesthesia, and the elbow joint was abducted in the supine position. Routine disinfection was performed and a tourniquet was applied to the proximal 1/3 of the upper arm. Approximately 5 cm above the elbow crease, an “*S*” incision was made from the medial or lateral border of the biceps muscle to the midline of the forearm, avoiding a 90° angle with the elbow crease and preserving intact soft tissue structures as much as possible.

When two or more fractures were involved, the skin incision was extended appropriately, after which the subcutaneous tissue was bluntly separated, identifying and protecting the medial cutaneous nerve of the forearm (identifying the lateral cutaneous nerve of the forearm when a lateral incision was used), basilic vein, cephalic vein, and median cubital vein.

**Fig. 1:**
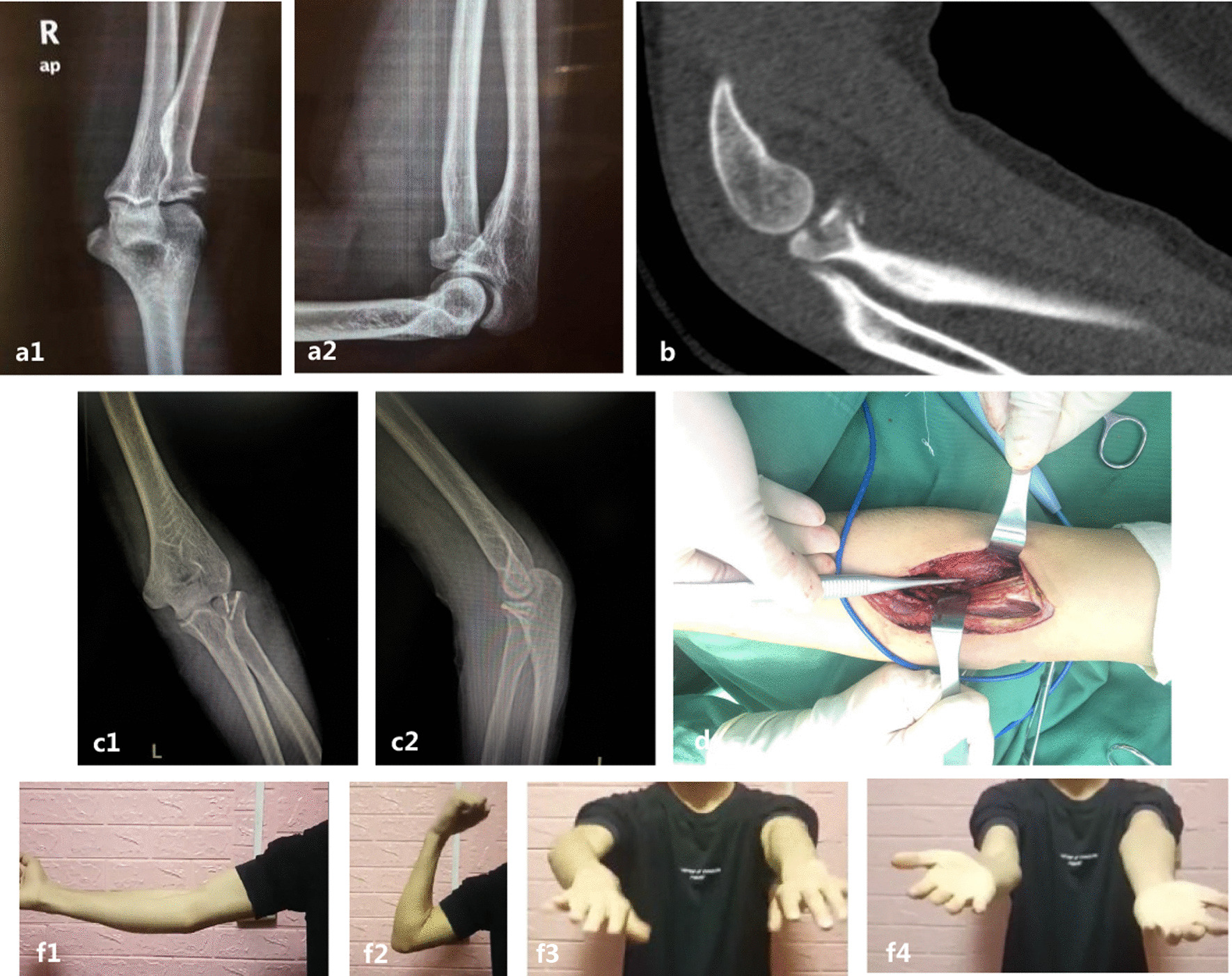
A 23-year-old man presented with a type of mason: II radial head fracture. Preoperative X-ray (**a**) and computed tomography (**b**). Intraoperatively, the biceps brachii was exposed and pulled medially to enter between the brachioradialis and biceps brachii interval, with the supinator muscle was located in its deeper layers (**d**). Solid union and good outcomes were achieved at the 1-year follow-up (**c, f**)

1. To manage a coronoid fracture, the bicipital aponeurosis was lifted with a vascular clamp and then incised. The biceps muscle was pulled laterally, and the pronator teres muscle was pulled medially, with the brachial artery and median nerve in its deeper layers and the median nerve medial to the brachial artery. At this point, the elbow joint was slightly flexed by 5°–10° to keep the vascular nerve bundle in a tension-free state, and the brachial muscle was exposed in the interval between the brachial artery and the median nerve. The brachial muscle was incised along the course of the muscle fibers to expose the joint capsule.
2. In the management of radial head fractures, the biceps brachii muscle was first pulled medially (Fig. 1d). The forearm was fixed in a state of extreme supination, the supinator muscle was cut at the insertion point where the supinator muscle attaches to the radius while avoiding the Frohse arch, and finally, the joint capsule was opened to expose the radial head.
3. Both of these intervals were used to expose the distal humerus by extending proximally, but a closer interval was selected depending on the type of distal humeral fracture.

Most fracture fragments reset themselves after the joint capsule is opened and the elbow joint fully extended. A 1.5-mm Kirschner wire was typically used to temporarily fix the fragment in the anteroposterior direction and maintain pressure on it. After fluoroscopy showed that the fracture fragments were satisfactorily repositioned, the appropriate internal fixation device was selected according to the fracture type and fixed after shaping the fracture according to its morphology.

In patients with elbow dislocation, the elbow was examined for stability after reduction, and a concentric reduction was achieved, with an arc of flexion–extension from 20° to 130° and no posterior or posterolateral subluxation or dislocation. Elbows that could not maintain stability required ligament repair or reconstruction, and if necessary, a combined approach was used. The tourniquet was released before closure, and meticulous hemostasis was performed. Drainage was placed, and the incision was sutured and dressed.

### Postoperative management

Patients were supplemented with a hinged plastic brace for postoperative immobilization. The forearm was positioned in a pronation or supination position in cases of lateral or medial collateral ligament deficiency, respectively. The forearm was splinted in neutral rotation if both the medial and lateral collateral ligaments had been repaired. A light diet was offered at 2 h after the operation. Regarding the use of antibiotics, we routinely administer cefuroxime sodium (3.0 g) intravenously once at 30 min^−1^ h before surgery, with an additional postoperative dose of antibiotics if the operation is longer than 2 h. For patients with terrible triad injury, which is difficult to restore, we applied antibiotic once intraoperatively if the operation was more than 3 h.

The patients were given intravenous cimetidine, and the affected limbs could be elevated to reduce swelling and prevent stress after general anesthesia. Analgesic treatment was actively administered according to the VAS score, and single or combined analgesic drugs were used depending on the patient's condition; the purpose was to facilitate rest and recovery and thus promote healing to effectively reduce the length of hospitalization and the patient's medical expenses. Anteroposterior and lateral elbow films were reviewed the day after surgery. The dressing applied was changed regularly, and drainage was usually removed within three days postoperatively. On the second postoperative day, passive flexion and extension exercises of the elbow were started. Active movement of the wrist and metacarpophalangeal joints can help determine whether a nerve has been injured during surgery, and elbow flexion and extension and rotation exercises were gradually performed beginning at 1 week after the operation. Active flexion and extension exercises began in the 2nd week. Unrestricted flexion and extension and strength exercises were performed in the 8th week. In addition, to prevent heterotopic ossification, oral nonsteroidal anti-inflammatory drugs such as indomethacin for six weeks were recommended, and those with severe gastrointestinal reactions were advised to use etoricoxib tablets.

### Data collection and analysis

**Fig. 2:**
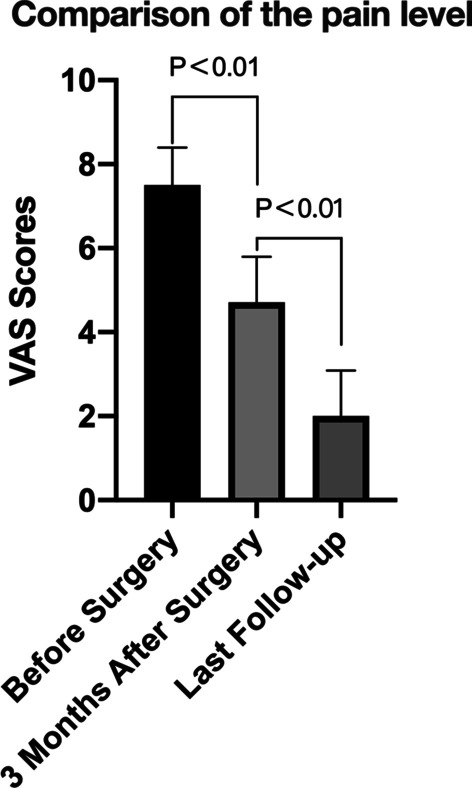
The VAS scores of patients before surgery, at 3 months after surgery, and at the final follow-up were compared

For follow-up, clinical and radiological examinations were performed by a clinical investigator who was not involved in the treatment. The clinical evaluation included the patient's bilateral arc of motion in elbow flexion, extension, pronation, supination, Mayo Elbow Performance Score (MEPS) [12], and examination of detectable surgical complications. The VAS pain scores of the patients before the operation, three months after the operation, and at final follow-up were compared (Fig. 2). Postoperative X-rays were evaluated for fracture union, implant loosening, heterotopic ossification, degenerative changes, and joint congruency. Intraoperative and postoperative complications were also recorded. A radiological fracture union was obtained when the fracture showed evidence of external callus bridge across the fracture line in the three cortices on the lateral view of the elbow. Statistical analysis was performed using SPSS version 26.0 software (SPSS Inc., Chicago, IL, USA).

## Results

All coronal plane fractures were successfully treated with internal fixation using an anterior approach, and final follow-up X-rays showed radiological fracture union. The average time to radiologic union was 14.4 weeks (range, 12–18 weeks). Intraoperatively, the mean surgical incision length was 8 ± 2 cm and the mean operative time was 123 ± 59 min.

**Fig. 3:**
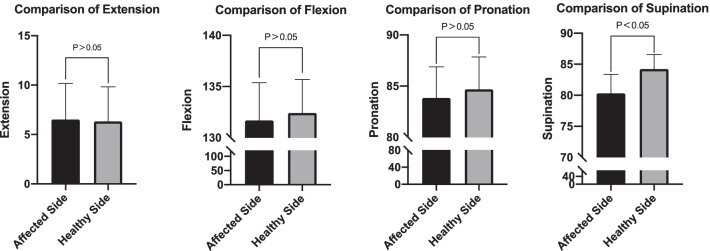
Comparing the mobility of the affected and healthy elbow joints after surgery

Outcomes are provided in Table 2. The mean duration of follow-up was 21 months (range, 12–36 months). At the final follow-up, the mean elbow flexion arc was 129 ± 7°, and the extension arc was 9 ± 6°. The mean pronation arc was 83 ± 3° and the supination arc 80 ± 3°. The mean MEPS was 90 ± 8 points, with 18 and 20 cases of excellent and good results, respectively. There was no significant difference in elbow extension, flexion, or pronation between the 31 cases of single fracture and the healthy side (*P* > 0.05), but the arc of supination was slightly worse than that of the healthy side (*P* < 0.05) (Table 3; Fig. 3). Pain was significantly reduced after treatment (*P* < 0.05) (Table 4).

**Table 2:** Follow-up information

Case	Follow-up (mo)	Arc of motion								MEPS
		AE	HE	AF	HF	AP	HP	AS	HS	
1	18	11	10	138	140	82	83	80	82	100
2	24	16	7	120	135	83	87	80	84	95
3	17	8	8	134	136	82	85	81	83	100
4	22	10	10	130	130	80	82	79	84	85
5	23	11	10	132	134	80	81	79	80	95
6	12	12	10	128	129	82	84	80	83	85
7	22	0	0	135	135	85	83	80	82	100
8	12	11	10	132	130	83	83	83	83	80
9	38	4	3	128	132	84	84	82	87	95
10	10	0	0	134	135	88	89	85	87	100
11	12	18	0	123	136	82	89	80	82	100
12	20	0	0	137	137	90	87	77	82	85
13	14	7	8	127	128	80	83	79	85	80
14	12	0	0	126	128	83	84	82	87	85
15	25	7	7	128	130	81	81	76	84	100
16	35	11	11	134	135	81	82	78	85	85
17	33	9	8	125	128	88	90	78	84	85
18	36	25	8	105	129	80	90	76	79	85
19	30	4	4	135	135	87	90	78	88	95
20	26	17	9	124	135	82	89	79	89	95
21	25	19	7	120	136	82	83	80	90	85
22	19	18	11	128	133	83	85	76	88	95
23	19	22	5	110	131	80	88	81	90	75
24	18	0	0	136	137	86	86	83	85	85
25	26	9	10	127	127	84	80	76	85	80
26	28	6	6	128	131	80	87	81	81	85
27	23	9	9	139	132	89	86	78	82	85
28	26	5	5	134	135	85	82	78	82	100
29	18	6	6	131	132	80	81	77	82	80
30	17	4	4	133	133	85	86	84	83	85
31	19	9	8	127	129	80	81	76	84	85
32	21	8	9	132	133	82	86	80	85	85
33	15	7	6	132	135	87	90	82	89	90
34	21	9	9	133	133	83	79	82	83	85
35	21	8	7	128	128	85	86	79	83	100
36	14	5	5	132	133	85	88	85	86	95
37	17	7	8	133	135	83	87	83	85	100
38	19	5	5	134	130	89	89	89	90	95

*mo* month, *AE* affected side extension degree, *HE* healthy side extension degree, *AF* affected side flexion degree, *HF* healthy side flexion degree, *AP* degree of affected pronation, *HP* degree of healthy pronation, *AS* degree of affected supination, *HS* degree of healthy supination, *MEPS* Mayo Elbow Performance Score

**Table 3:** Comparison of the movement arc of the healthy side and the affected side in 31 cases of a single fracture

	Healthy side	Affected side	*p*
Flexion°	132.42 ± 3.3	131.68 ± 3.7	0.058
Extension°	6.32 ± 3.5	6.52 ± 3.7	0.136
Pronation°	84.68 ± 3.2	83.84 ± 3.0	0.072
Supination°	84.23 ± 2.4	80.32 ± 3.1	0.001

**Table 4:** Comparison of VAS scores of patients before and after surgery

	Before surgery	3 Months after surgery	Last follow-up
$\overline{x}$	7.5 ± 0.9	4.7 ± 1.1	2.0 ± 1.1
*p*	< 0.01	< 0.01	< 0.01

**Fig. 4:**
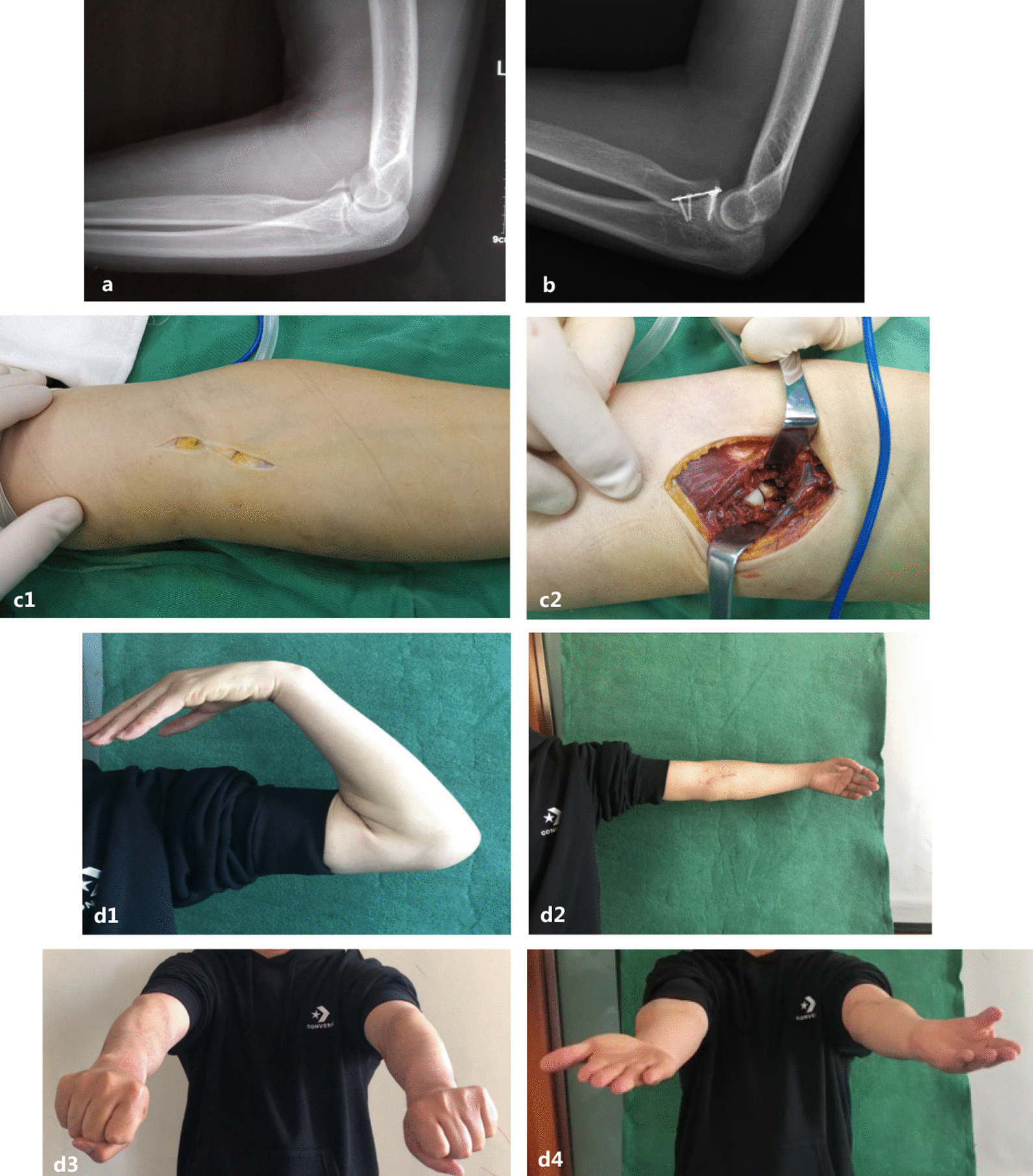
A 40-year-old man presented with a Regan and Morrey type II coronoid process fracture. Preoperative radiographs (**a**). Intraoperative exposure of the coronoid process through the anterior elbow approach (**c**). Solid union and good outcomes were achieved at the 8-month follow-up (**b, d**)

**Fig. 5:**
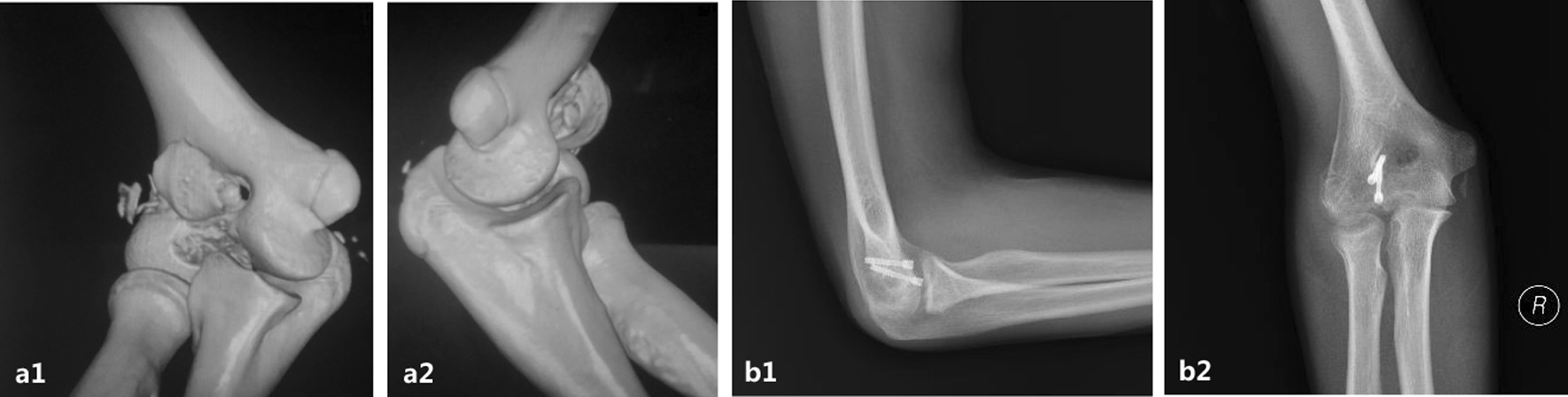
A 16-year-old boy diagnosed with AO type 13B3.2 humeral trochlear fracture (**a1, a2**). At the final follow-up, plain roentgenograms showed good bone union with good function (**b1, b2**)

**Fig. 6:**
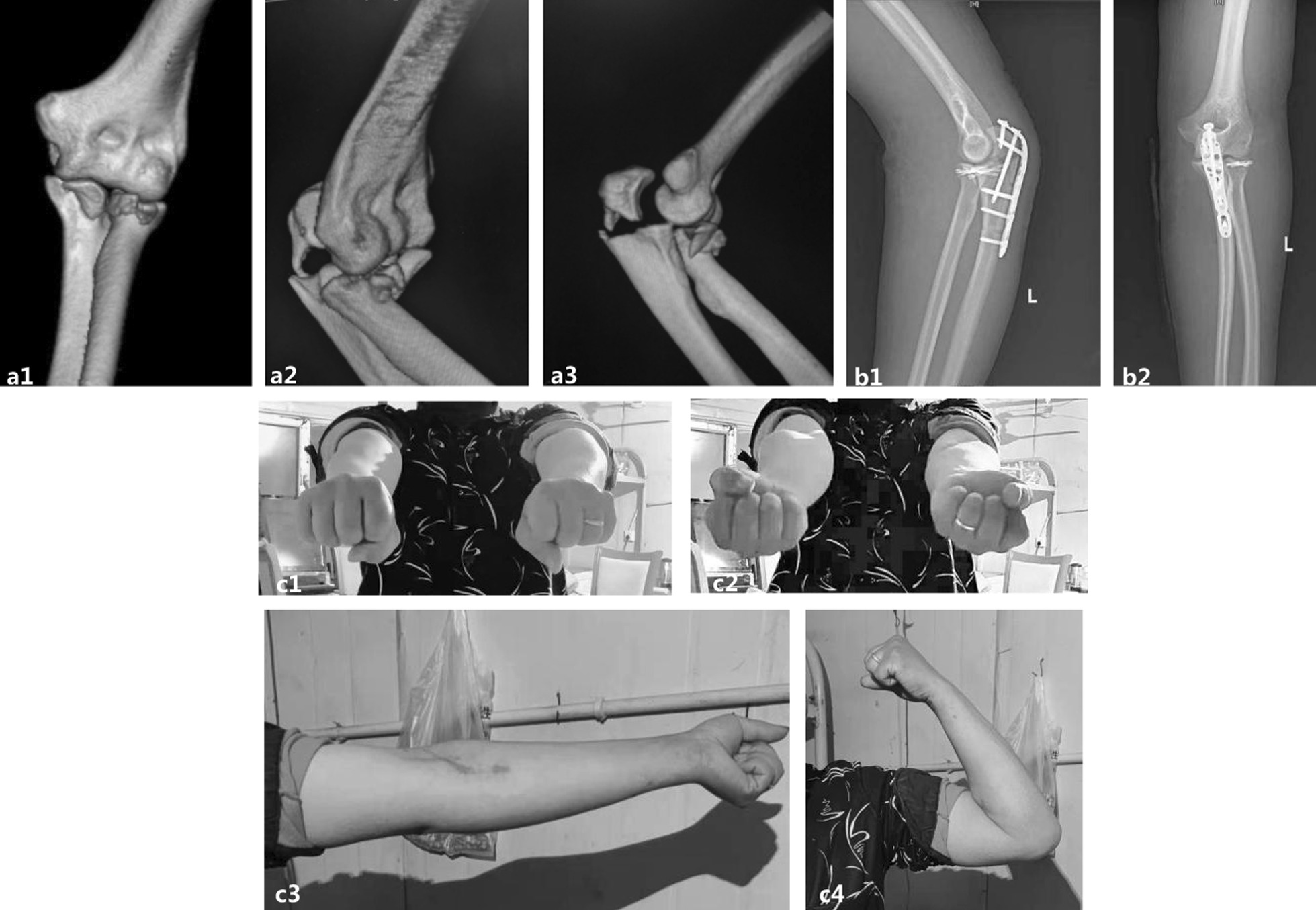
A 37-year-old man presented with a terrible triad of the elbow and an ipsilateral olecranon fracture. Preoperative CT 3D reconstruction (**a**). Postoperative plain radiographs at one month showed strong internal fixation and good alignment of the fracture (**b**). Good outcome was achieved at the 1-year follow-up (**c**)

There were no intraoperative complications. There were also no wound complications or implantation failures. Two patients experienced transient postoperative median nerve paralysis; these symptoms disappeared after regular oral administration of nutritional nerve drugs and did not recur in subsequent follow-up. Two patients experienced transient postoperative median nerve paralysis, the symptoms of which disappeared after regular oral administration of nutritional nerve drugs and did not recur in subsequent follow-up. One patient exhibited mild heterotopic ossification and was not treated because it did not affect the function of the elbow joint. All patients returned to work and were satisfied with the treatment (Figs. 1, 4, 5, 6).

## Discussion

The anterior approach has been widely employed in upper limb surgery. It is considered a valuable option in the treatment of proximal radius fractures, reconstruction of the distal biceps tendon, resection of anterior elbow tumors, and soft tissue infection [13]. After the occurrence of elbow fractures, the bone fragments often dislocate to the coronal plane according to the mechanism of injury. However, due to the anatomical characteristics of the anterior elbow joint, surgeons often avoid the anterior approach due to fear of damage to the neurovasculature and often choose the lateral approach, which makes it difficult to directly view and address the fracture surface [14, 15].

Elbow joint fracture is an intra-articular fracture, and anatomical reduction of the articular surface is key for achieving strong fixation and a satisfactory elbow joint [16, 17]. Overall, adequate exposure of the articular surface is a prerequisite for anatomical reduction of fractures. Some authors [9] have compared the exposure area of the distal humerus articular surface by different surgical approaches and found that the average percentages of exposed articular surfaces by anterior and posterior olecranon osteotomy and medial and lateral approaches are 45.7% ± 2.0%, 53.9% ± 7.1%, 20.6% ± 4.9% and 28.5% ± 6.3%, respectively. They conclude that anterior and posterior approaches are preferable to medial and lateral approaches for intraoperative articular surface exposure in the treatment of distal humerus fractures. Yang et al. [10] compared the exposure area of coronoid process fracture between the biceps tendon-brachial artery interval (B-B interval) and brachial artery-median nerve interval (B-M interval) from an anterior approach; based on the results, the average exposed surface area of the coronoid process was 2.26 times greater with the B-M interval than with the B-B interval, allowing for visualization for fracture reduction.

Compared with other approaches, the anterior approach has some advantages, as follows [1, 7, 10]: (1) providing excellent visualization and more direct access to the articular surface (Fig. 4c); (2) allowing for anatomic reduction of the fracture and more fixation options, as anterior to posterior compression is more mechanically appropriate, reducing the risk of fracture fixation failure (Fig. 4b); (3) avoiding a large amount of soft tissue dissection; (4) avoiding damage to the flexor-pronator muscle mass and the ulnar nerve; and (5) exploring the ulnar collateral ligament of the elbow joint and repairing the anterior joint capsule, which is beneficial to the stability of the elbow joint and reduces the occurrence of heterotopic ossification.

As most elbow joint coronoid fractures are displaced forward, anterior surgery can be considered if the elbow joint is unstable [18, 19]. The Kocher approach is commonly used in radial head treatment [20], which is accessed from the extensor carpi ulnaris and the anconeus interval. It is primarily indicated for the repair of radial head fractures and lateral collateral ligaments, but there are a certain number of patients who present a combined coronoid fracture with a radial head fracture [21]. In such cases, the use of the lateral approach is obscured by the radial head, narrowing the operating space. Another surgical option in the treatment of the coronoid fractures is the osteotomy of the radial head, that could cause elbow joint instability and additional trauma. As the radius itself has intraoperative rotatable properties, even for radial head fractures that occur laterally in the sagittal plane, the fracture surface can be fully exposed when using the anterior approach.

The posterior approach provides the greatest exposure of the distal humeral articular surface in surgery for distal humerus fractures. However, common complications of this approach are nonunion, in 30% of patients [22, 23], a larger surgical incision and prolonged operation time. Due to the presence of an anatomical structure in the distal humerus that is tilted approximately 30° anteriorly, the advantage of the anterior approach allows for internal fixation under direct vision [9]. Therefore, the anterior approach is recommended for AO type B fractures that mainly occur on the coronal plane, including capitulum, trochlea, or combined fractures.

Another possible indication for anterior approach is the terrible triad of the elbow, a traumatic injury pattern of the elbow characterized by elbow dislocation, radial head fracture, and a coronoid fracture often associated with a collateral ligament injury. Traditional treatment usually includes a combined medial–lateral approach. In terms of the injury mechanism, an anatomical reduction under direct visualization via the anterior approach offers advantages that other approaches cannot. Some of the cases included in this study involved terrible triad injury, and all were treated by the anterior approach, with good results (Fig. 6).

The neurovascular complications, described by some authors, did not occur in our series. Only two patients experienced transient postoperative median nerve paralysis, and the symptoms disappeared after regular oral administration of nutritional nerve drugs and did not recur in subsequent follow-up. There was only one case of mild heterotopic ossification in the front of the elbow joint, which did not cause subjective symptoms to the patient or affect movement of the elbow joint during follow-up, and it was therefore not treated. There are concerns [14] that injuring the brachialis will increase the incidence of heterotopic ossification. Because there was no additional osteotomy, as with the posterior approach, we did not observe nonunion. A considerable number of the patients with postoperative limitation of motion experience fear, and providing active rehabilitation guidance to such patients after surgery is effective. The patients in this study showed significant improvement in the arc of motion after active rehabilitation instruction.

Based on the study results and long-term clinical practice, we summarize some noteworthy points regarding the anterior approach for the treatment of fractures of the elbow in the coronal plane.

1. Intraoperative elbow flexion of 5°–10° is beneficial to reduce muscle tension while allowing the sliding of vascular nerves between loose tissues and reducing the risk of vascular nerve injury.
2. Because the deep fascia below the external epicondyle of the humerus penetrates the lateral antebrachial cutaneous nerve, the lateral side of the incision should be within 1.5 cm of the outer edge of the biceps tendon. It may be necessary to cross the cubital crease. In such a case, the surgical incision should be selected in the position above the cubital crease to prevent the possibility of postoperative scar contracture affecting elbow joint activity, as the lateral antebrachial cutaneous nerve in this area is located in the deep layer and is not easily injured during surgery.
3. In the treatment of coronoid fractures, interval access between the brachial artery and the median nerve is chosen for the anterior approach; however, interval access between the median nerve and the pronator interval has a high risk due to the nerve branches present. The advantages of this approach are that it is safer to enter from the vascular-neural interval, it directly exposes the fracture site and facilitates reduction, and it facilitates fixation with a plate and causes less tissue damage; the disadvantage is that the medial coronoid process cannot be fixed.
4. Care needs to be taken when exposing the radial head, over which a deep branch of the radial nerve migrates across the Frohse arch as the posterior interosseous nerve. To avoid injury to the radial nerve, the radius needs to be rotated extremely posteriorly, the termination point of the posterior rotator muscle needs to be separated, and the joint capsule should be pushed from the inside out with an osteotome. Simultaneously, using the characteristics of the radius, the radial head fracture area is rotated toward the field of view for exposure.
5. Access to the bicipital tendon is determined by the type of fracture. For coronoid fractures and trochlea fractures located on the ulnar side, we used the medial interval, while for humeral capitellum fractures and radial head fractures, we used the lateral interval to access the bicipital tendon.
6. Intraoperative placement of drainage is essential, as adequate drainage prevents hematoma formation and reduces the risk of infection and heterotopic ossification.
7. Early rehabilitation activities are recommended. In particular, the elbow joint should not be overly bandaged, as a thick sterile dressing will greatly affect the arc of elbow bending.
8. To prevent heterotopic ossification after surgery, it is recommended that patients take oral nonsteroidal anti-inflammatory drugs such as indomethacin for six weeks; those with severe gastrointestinal reactions should take etoricoxib instead.

There were a few limitations in this study. The study was essentially retrospective, and there was no control group for comparison. Furthermore, the sample size of the different fracture types included was small, and we did not analyze combined fractures separately from simple fractures. Finally, studies on learning curves have not been conducted, and more in-depth analyses need to be carried out. Future work should also explore the efficacy of the anterior approach in the treatment of terrible triad injury based on prospective studies with expanded sample sizes.

## Conclusion

The anterior approach for the treatment of coronal plane elbow fractures, especially combined fractures, provides a clear surgical exposure, large operation space, easy reduction, more fixation options, less trauma, fewer complications, and good postoperative function recovery. In conclusion, the anterior approach is reliable in the management of coronal plane elbow fractures and deserves to be implemented in clinical practice.

## Data Availability

The datasets analyzed during the current study are available from the corresponding author on reasonable request.

## Abbreviations

MEPS: The Mayo Elbow Performance Score
VAS: The visual analogue scale scores
B-B interval: The biceps tendon-brachial artery interval
B-M interval: Brachial artery-median nerve interval
